# National ownership of family planning: What do FP2020 commitments have to do with it?

**DOI:** 10.12688/gatesopenres.13225.1

**Published:** 2021-02-24

**Authors:** Gabrielle Appleford, Priya Emmart

**Affiliations:** 1 AVENIR, Nairobi, 00606, Kenya; 2 AVENIR, Glastonbury, Connecticut, USA

**Keywords:** FP2020, family planning, national commitment

## Abstract

**Background: **In 2020, we reached a family planning (FP) temporal milestone. This paper seeks to understand the political economy of commitments and normative best practice within FP national programs, contributing to “stock taking” of change objectives for national ownership and domestic financing of FP programs post FP2020. Stock taking is needed to understand, for example, do we expect our current approaches to deliver greater commitment or do we need to change our approach? Is time the limiting factor for FP2020 commitments or are other, contextual, mechanistic and implementation factors more critical?

**Methods: **This paper uses mini-case studies to offer insights in response to these questions. It drew from country status updates of national FP program commitments published on the FP2020 website. These included country self-assessments, country action for acceleration plans and revitalised commitments using standard templates provided by FP2020.

**Results: ** Critical factors emerging from the case study analysis suggest the following. *Context*: Country programs that adapted best practices through thoughtful selection, regular monitoring, and course correction, were more responsive to context and better able to scale interventions. *Mechanism*: Programs that embedded commitments within national health reforms and transformative agendas were able to sustain commitment and mechanism more effectively over time. *Implementation*: Programs that were able to balance central coordination with devolved implementation, more effectively translated commitments to action. *Monitoring*: Programs that placed emphasis on monitoring progress and course correct were better able to steward national commitments and partner inputs.

**Conclusions: **National FP programs included within the country comparative analysis benefitted from their engagement with FP2020. However, not all were able to convert FP2020 commitments into national ownership. In many FP2020 contexts, there is less need for a technical intervention and greater need for engaging politically on sensitive issues that constrain women’s and adolescent empowerment and rights and access to FP.

## Introduction

In 2020, we reached a family planning (FP) temporal milestone. We also reached women. Have we reached them with impactful and sustainable programs? Coronavirus disease 2019 (COVID-19) is pressure testing national FP programs and the health systems that underpin these. As the virus becomes a part of our contextual fabric, it is timely to think about the durability of FP programs and their outcomes, to reflect on progress in reaching women with quality FP services, now and over her life course.

Reflection on FP2020 ( Box 1) country performance is typically done on an annual basis, as part of national family planning consensus meetings. Annual estimates include a standard set of 18 core indicators that capture additional FP use, the method mix and method availability, aspects of FP quality and equity, and estimate mortality averted. National programs also have more bespoke monitoring of commitments towards national ownership. While these are country defined, they tend to be *“highly similar across dissimilar contexts” ^1^*, as many country programs draw upon global best practices. Like the 18 core indicators, performance against commitments is reviewed on an annual basis.

**Box 1. FP2020 overview**

FP2020 is a global partnership that supports the rights of women and girls to decide, freely and for themselves, whether, when and how many children to have. FP2020 works with governments, civil society, multi-lateral organizations, donors, the private sector, and the research development community to enable 120 million more women and girls to use contraceptives by 2020.

Source: FP2020 ( http://www.familyplanning2020.org/)

This paper seeks to understand the political economy of commitments and normative “best practice” within FP national programs. The paper contributes to “stock taking” of change objectives for national ownership and domestic financing of FP programs for next phase of FP2020. Stock taking is needed to understand, for example, do we expect our current approaches to deliver greater commitment or do we need to change our approach? Is time the limiting factor for FP2020 commitments or are other, contextual and mechanistic factors more critical?

## Methodology and analytical framework

### Methodology

The paper developed country case studies to understand the political economy of national FP2020 commitments. It drew from country status updates of national FP program commitments published on the FP2020 website. These included country self-assessments, country action for acceleration plans and revitalised commitments using standard templates provided by FP2020. These were completed by national FP program managers with support of partners. Partners included USAID and UNFPA FP2020 focal points who participated in specific FP2020 regional workshops where country commitments were reviewed and at times, revised. During the 2018–2019 period, some country programs also underwent FP2020 high impact practices (HIP) analyses where commitments were categorised based on HIP, and cross checked against national commitments, national FP costed implementation plans (FP-CIPs) and updates provided through self-assessments and plans. Some other resources supplemented the FP2020 reports. These were either posted on the FP2020 country commitment page as supplemental documents or sourced by the authors. All quotes in the case studies (where not otherwise referenced) are from the FP2020 website and draw on FP2020 national documentation (they are referenced using the reporting year) ^i^.

The country case studies were selected to offer insights into commitment making and implementation. These follow a timeline from initial FP2020 commitment – most dating from 2012 – through to 2019/20. A subset of FP2020 countries were selected by the authors based on:

- **Geographic context** – diversity in geographic, demographic, and linguistic contexts.
- **Program context** - low, medium, and high prevalence mCPR program contexts.
- **Financing context** – inclusion of countries with high dependence on donor financing and those transitioning to domestic financing.

Representation was sought across FP2020 contexts to illustrate the range of geographic, programmatic and financing contexts. Program contexts are summarized using S-curve categories – low mCPR, low growth (Niger); low mCPR but growing (Senegal, Burkina Faso and Afghanistan); growth and high potential for growth (Pakistan and Rwanda); plateauing growth with population inequities (Kenya, India and Malawi). A simple key for financing has been employed, with “D” signifying donor and “T” signifying “transition” to domestic financing. The case studies have been ordered to emphasize inter-case differences. A summary of the case studies is included in Table 1.

**Table 1.** Country geographic, programmatic and financing contexts.

Geographic context	East and southern Africa				West Africa (francophone)			South Asia		
Country	Kenya	Ethiopia	Malawi	Rwanda	Senegal	Burkina Faso	Niger	Afghanistan	India	Pakistan
Program context	Plateau	Growing	Plateau	Growing	Low but growing	Low but growing	Low	Low but growing	Plateau	Growing
Financing context	D - T	D - T	D	D - T	D	D	D	D	T	D

D = donor; T = transition.

### Analytical framework

Our analytical framework considered context, mechanism of impact, and implementation ^2^. Box 2 aligns our understanding of these concepts with FP2020 concepts.

**Box 2. Alignment of concepts**

FP2020 uses three broad categories of commitment: program; policy and political; and financial. Within our analysis, we use mechanism for programmatic commitment while financial, policy and political are analysed as part of context and implementation.

- **Contextual factors** – factors external to the intervention which may influence its implementation, or whether its mechanisms of impact act as intended.
- **Mechanism of impact** - the intermediate mechanisms through which intervention activities produce intended (or unintended) effects.
- **Implementation** - the process through which interventions are delivered, and what is delivered in practice.

An extraction template was developed that listed countries on one axis and document year on the other axis. This allowed for chronological extraction of FP2020 documentation to understand progress and changes in commitments over time. We extracted for mechanisms of impact (i.e., the key policy reforms or programmatic interventions proposed by country commitment makers), implementation status and contextual factors that might aid or impede progress (such as insecurity in some Sahelian contexts and devolution in the Kenya context).

## Country case studies

### Kenya: *National commitment demonstrates fluctuations in mechanisms and changes in context*

**Contextual factors**: Devolution, which commenced from 2012/13, negatively affected domestic financing of FP commodities, concurrently with a reduction in donor financing and alongside other “vertical” priority program transitions ^3^.

**Mechanisms:** Different mechanisms to address national commitments were proposed over time through FP2020 action plans and self-assessments. There was evidence of partner influence on proposed mechanisms, such as total market approach (TMA) and sub-national FP-CIPs. These mechanisms did not effectively address commodity security, which deteriorated over the life course of FP2020.

**Implementation:** There was a lack of implementation and/or monitoring of some mechanisms. There was a lack of effective inclusion of FP within some mechanisms, such as the National Hospital Insurance Fund (NHIF) and maternal and child health (MCH) “entitlement” programs. 

**Figure 1.**
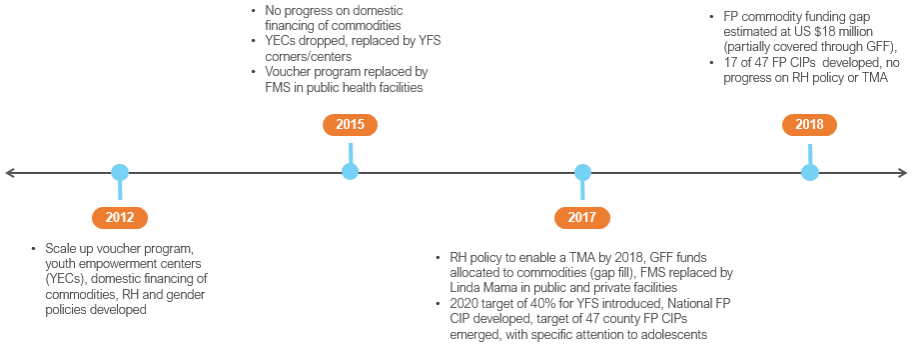
Kenya FP2020 commitments and timeline. YECs – youth empowerment centres; RH – reproductive health; YFS – youth friendly services; FMS – free maternity services; TMA – total market approach; GFF – Global Financing Facility; CIP – costed implementation plan.

In 2012, Kenya committed to scale up the output-based aid (OBA) program, a Government of Kenya (GoK) and German collaborative, that included FP, amongst other reproductive health (RH) services ( Figure 1) ^ii^. There was expressed intent to also address youth through the scale up youth empowerment centers (YECs), as a “one-stop-shop” for youth friendly information, including contraception. Kenya committed to continued domestic financing of commodities. Predating FP2020, the national FP program had progressively contributed to commodities, growing its contribution from US $2.5 million for the period 2005–2006 to US $6.6 million for the period 2012–2013 ^4^. While there was a recognised funding gap of 60 per cent in 2012, the program sought to fill this with donor resources. The year 2012 also coincided with a new constitutional dispensation, that transferred resources and responsibility for health service delivery to the country’s 47 counties.

In 2015 the Kenya FP2020 Secretariat registered a “temporary loss” of the FP budget line following devolution. This was reported alongside increasing commodity requirements, given the high mCPR in the country. Sub-national (county) FP-CIPs were introduced as a means of galvanising county resources for FP. The FP2020 country action plan of 2016 highlighted challenges with the financing gap for commodities and continued to attribute this to devolution. It also highlighted *“inefficiency and coordination challenges amongst partners”*, which made it difficult to fully map available FP resources and signalled possible duplication of resources ^5^. Commodity security remained a priority, YECs were dropped, and in their place youth friendly services (YFS) through corners and standalone centres promoted. The OBA program was replaced by the government’s Free Maternity Scheme in public health facilities; of note, this only nominally included FP as part of post-natal care (PNC).

The year 2017 heralded the launch of a new national FP-CIP for the period 2017–2020. While no review of the previous FP-CIP was done as part of this, it was acknowledged that the FP-CIP for 2012–2016 *“was not optimally utilised as had been envisioned at the time of its development mainly due to constitutional changes that saw devolution implemented.”* ^6^ New FP2020 actions for acceleration emerged including partnership with the private sector through a TMA with commitment to a revised RH policy to enable a TMA by 2018. While there was no progress on domestic financing, World Bank/the Global Financing Facility (GFF) funds were allocated to address the ever-increasing commodity financing gap. From 2017, FP commodities were to be costed before distribution to counties with an aim of increasing allocation for these at the county level. The Free Maternity Scheme was replaced by the NHIF “Linda Mama” maternity entitlement program, implemented in public and private facilities. Again, this only nominally addressed FP as part of PNC ^7^. A 2020 target of 40 per cent for YFS was introduced from a 10 per cent baseline (it was not made clear how this baseline was established). A focus on the development of 47 county FP-CIPs also emerged, with specific attention to adolescents.

In 2018, the FP commodity funding gap was estimated at US $18 million. The action was to continue to advocate with partners while US $5 million was allocated from the GFF kitty. FP continued to be notionally included within the NHIF and plans for universal health care (UHC). It was reported that 17 of 47 FP CIPs were developed; however, their influence on county resources for FP was not ascertained. It was also reported that there was increased national stewardship for TMA, supported by partners to institutionalize the approach: *“a FP2020-aligned TMA plan is being drafted which will guide the various partners over the coming years, and is based on the FP 2020 commitments.”* In practice, TMA does not appear to have gained traction within the Ministry of Health; as of Aug 2020, validation and launch of the TMA plan for FP was reported as “stalled” ^8^.

In 2019, the main driver of commodity insecurity continued to be the growing financing gap. This gap has widened over time due to a reduction in donor *and* domestic financing as well as an increasing total number of modern method users, driven by Kenya’s youthful population. In the absence of county resources for FP, responsibility for financing of commodities was returned to the national level, spurred by a donor-led match fund, and sliding scale proposal ^3^. As part of this, the GoK released US $10.2 enabling them to procure, warehouse and distribute contraceptives with plans to release further tranches in future. However, transition to national domestic financing of FP commodities is taking place alongside other program financing transitions, notably HIV/AIDS ^3^.

FP2020 national commitments since 2012 did not drive strategic preparation for transition from donor funding or replacement with domestic resources. Domestic resource mobilisation was considered a “best practice” in 2012 when the GoK financed 40 per cent of its contraception commodities; however, this practice was eroded over the period 2012-2019 due to a change in context and mechanism. Kenya’s FP2020 commitments may have signalled recognition of affordability and financing concerns but did little to address these in practice. FP stewardship at national level was also eroded. 

*“Governmental devolution, which commenced in 2013, has done little to correct for FP disparities and may have diluted technical support for FP, as partners have concentrated efforts and resources at the county level without commensurate engagement at the national level, where FP is technically stewarded.” ^3^*

### Afghanistan: *National commitment demonstrates strong management of the context and implementation*

**Contextual factors:** Afghanistan is a fragile state, which is heavily donor and partner dependant. Given this, the national FP program has been prone to partner influence.

**Mechanisms:** The Government of Afghanistan (GoA) ground national FP2020 commitments in policy and strategy. It invested in mechanisms for program review. As part of this, it was the first country to include the estimated modern use (EMU) embed in the DHIS2.

**Implementation:** The FP program demonstrated strong use of data and evidence. There was also strong management of partners and their alignment to the national FP/RH policy and strategy through regular engagement and review of commitments.

**Figure 2.**
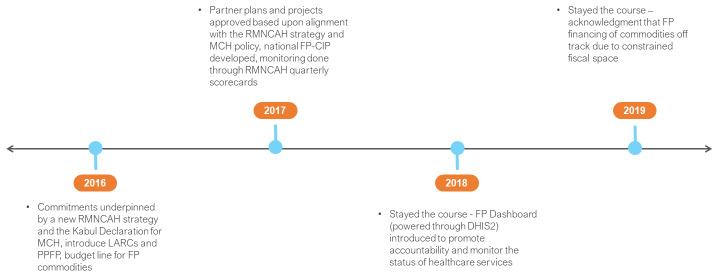
Afghanistan FP2020 commitments and timeline. RMNCAH – reproductive, maternal, newborn, child and adolescent health; MCH – maternal and child health; PPFP – post-partum FP; CIP – costed implementation plan; DHIS2 – District Health Information System 2.

Afghanistan became a FP2020 commitment maker in 2016 ( Figure 2). An assessment published from the same year indicated that the national FP *“oversight and intelligence role appears to be weak or virtually non-existent, that…decision-making and development of strategies are not evidence-based.”* ^9^ Commitments redressed these concerns, underpinned by a new reproductive, maternal, newborn, child and adolescent health (RMNCAH) strategy (2017–2020) and the Kabul Declaration for MCH (2015). To expand access, the national FP program committed to the introduction of long acting reversible contraceptives (LARCs), their inclusion on the essential drug list (EDL) and implementation strategies such as post-partum FP and private sector engagement. To guide programmatic decision making, quarterly reviews of the RMNCAH scorecard were initiated. Despite a limited domestic resource base, the GoA committed to the establishment of a budget line for FP commodities.

The GoA reaffirmed its strategic commitments through its actions for acceleration (2017) and consecutive self-assessment reports (2018 and 2019). A national FP-CIP for the period 2018–2022 was prepared. It was reported that partner plans and projects were approved based upon their alignment with the national FP-CIP, the RMNCAH strategy and MCH policy. To facilitate private sector participation, a RMNCAH minimum standard guideline was developed and 32 (of 40) memorandums of understanding (MoUs) signed with private hospitals and clinics for the provision of FP services. There was evidence of close monitoring of program and partner activities as each consecutive FP2020 report demonstrated progressive realisation of access targets. RMNCAH quarterly scorecards continued and a FP Dashboard (powered through DHIS2) was introduced to promote accountability and monitor the status of healthcare services. Self-assessment acknowledged that the domestic financing target was off track.

While the GoA showed strong central leadership and coordination of partner inputs, this was not picked up through a FP2020 HIP analysis, conducted in 2018. This reported no progress on several HIPs due to *“competing priorities”*, and *“weak intersectoral coordination”* amongst other challenges. However, the analysis suggests that the Afghani self-assessment reports - rich in data and other information – exemplify national commitment. Afghanistan’s fragile context and partner base were managed to good effect and are also exemplary, *“as members of the Family Planning Technical Committees, partners were involved in all decisions made by the Ministry of Public Health and other activities like information sharing and advocacy.”*

### India: *National commitment demonstrates transformative mechanism but with unanticipated effects of implementation*

**Contextual factors:** India’s history of limited choice in FP methods coupled with low empowerment and agency of poor and marginalized women and couples (i.e. based on caste, age) were the contextual backdrop to mechanisms to expand method choice.

**Mechanisms:** India committed to expand FP access and choice through Government of India (GoI) service delivery mechanisms and transition from donor to domestic financing, including from international to “indigenous” manufacture of FP commodities.

**Implementation:** There was demonstrated consistency in GoI commitments and mechanisms over time. However, some of these were not well institutionalized within the GoI or were dependent on partners. This included demand creation, behaviour change and quality assurance which limited the impact of mechanisms to expand FP choice.

**Figure 3.**
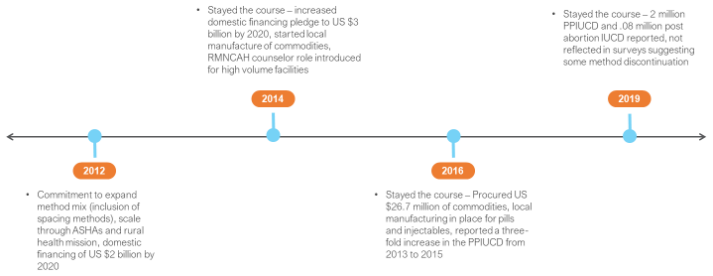
India FP2020 commitments and timeline. ASHAs – Accredited Social Health Activist; RMNCAH – reproductive, maternal, newborn, child and adolescent health; PPIUCD – post-partum intra-uterine contraceptive device; IUCD - intra-uterine contraceptive device.

In 2012, the GoI made large scale, national commitments that built from existing service delivery mechanisms and implementation modalities ( Figure 3). The centrepiece of India's FP2020 commitment was a deliberate shift from limiting to spacing methods, and an expansion of the choice of methods, that included intrauterine devices, pills and injectables. Previously, sterilisation had been the hallmark of India’s FP program, associated with demographic, target driven and at times, coercive practices ^iii, 10^. The proposed service mechanism provided scale and included 860,000 community health workers - Accredited Social Health Activists (ASHAs) - and 150,000 rural health sub-centres under the National Health Mission. The GoI backed commitment with domestic financing, with a pledge of US $2 billion by 2020.

In 2014, the GoI decided to *“up the ante”* by increasing its FP2020 pledge to US $3 billion by 2020. It also launched a national strategy to address the needs of adolescents, that included sexual and reproductive health (SRH), supported through the ASHA network. The GoI 2015 self-assessment reported that the manufacture of FP commodities was being done “indigenously” with commodities worth US $42.3 million procured each year; as reported, *“no donor funds are being sought for the same.”* For 2014–15 period, the financial outlay was reported to have increased by 45 per cent (from 2012). A new position of RMNCAH counsellor was created to ensure counselling for FP at high case load facilities as previously counselling had focused on limiting, not spacing children.

The GoI maintained its FP2020 commitment course in 2016. During the 2015–2016, FP commodities worth US $26.7 million were procured. The GoI reported indigenous capacity for manufacturing the injectable and oral contraceptives. To reinforce method choice, a media campaign was launched but experienced challenges with planning and budgeting at an early stage. Social franchising through partners was promoted in selected high need states to improve private sector participation, address service delivery gaps and demand generation. During this period, a three-fold increase in the post-partum intrauterine contraceptive device (IUCD) performance from 2013 to 2015 was reported, with a 65 per cent increase from 2014 to 2015.

In 2019, it was estimated that approximately 2 million post-partum IUCD insertions had been done since the inception of the program or 16 per cent of total public health deliveries. It was also estimated that 0.08 million post-abortion IUCDs had also been done, or 8 per cent of the total abortions in the public health system. Performance, however, was not reflected in surveys, which suggested high levels of method discontinuation (based on the low prevalence estimated from surveys).

The India case study exemplifies national commitment to programmatic transformation, through a deliberate shift from limiting to spacing methods. The GoI used existing platforms to overlay this programmatic objective, and provided technical solutions (e.g., training, commodity manufacturing) for its delivery. However, less emphasis was placed on the political economy of existing platforms and the agency of frontline workers, whose ingrained practices may not have been client or quality oriented. In a context in which women were given the “choice” to space their fertility, they could also choose to discontinue or switch methods. Women’s and couple’s increased agency may have been transformative in the context of a previous regime of sterilisation and limited choice but ran counter to mCPR targets.

### Pakistan: *National commitment demonstrates a mismatch between stated priorities at national level and mechanisms for implementation*

**Contextual factors:** There was limited engagement of federal policy makers and heavy reliance on partners and donors in a highly decentralised context with varying levels of FP programming across contexts (provinces).

**Mechanisms:** There was no identified central mechanism of impact; rather, mechanisms were left to the provincial population and development offices and partners to define. Efforts were made to gain policy consensus in response to uneven mechanisms of impact at provincial level.

**Implementation:** Weak national stewardship of the FP program over the life course of FP2020 resulted in limited ownership and uneven implementation of the FP program at provincial level. There have been efforts to correct this through the establishment of a Country Engagement Working Group.

**Figure 4.**
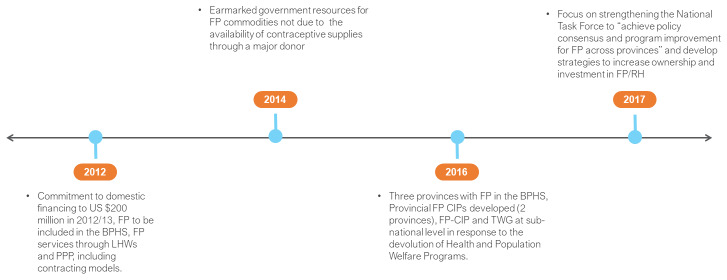
Pakistan FP2020 commitments and timeline. BPHS – basic package of health services; CIP – costed implementation plan; TWG – technical working group; FP/RH – family planning/reproductive health.

In 2012, the Government of Pakistan (GoP) focused on financial commitments, pledging to increase estimated FP spending from US $151 million in 2011/12 to US $200 million in 2012/13, with commitment to further increases in future years ( Figure 4). In 2014, it was reported that earmarked government resources for FP commodities would only be utilized once a major donor’s contraceptive supplies came to an end the following year. Programmatically, in 2012, FP was reported to be included in the basic package of health services (BPHS) in two of Pakistan’s four provinces and none of its two autonomous territories. Key strategies were the delivery of FP services through Lady Health Workers (LHWs) and public-private-partnership (PPP), including contracting models.

Over the course of 2015–2018, FP-CIPs were developed at provincial level for the four provinces (none of the territories). These all have different start and end dates, some of which extend beyond 2020. Provincial FP technical working groups (TWGs) were also constituted in a subset of provinces. FP-CIP and TWG emphasis at sub-national level was in response to the devolution of Health and Population Welfare Programs funded through the federal government. By 2016, a third province was reported to have included FP in the BPHS.

In 2017, the GoP revitalised its commitment, and focused on strengthening the National Task Force/National Population Commission on Population and Development to *“achieve policy consensus and program improvement for FP across provinces.”* There was also emphasis on federal coordination with the provinces to agree on specific service delivery targets with financing aligned with the achievement of these targets. By 2018, emphasis was again directed to the federal government to develop strategies to increase ownership and investment in FP/RH.

The Pakistan case study exemplifies a lack of uniform coordination and program mechanism with heavy reliance on partner-led implementation and donor financing. There was progressive recognition by the GoP of the need for national oversight given different provincial models, FP-CIPs and timeframes. This was also prompted by limited provincial investment in FP/RH.

### Ethiopia: *National commitment to improvement of central mechanism and reaching underserved populations*

**Contextual factors**: The Government of Ethiopia (GoE) stewarded growth in mCPR that pre-dated FP2020; however, inequities in regional and population access to FP remained. The GoE demonstrated strong interest in addressing the developmental priorities of youth and adolescents, including access to contraception.

**Mechanisms:** The national Health Extension Program (HEP) was positioned as the main mechanism for FP2020 commitments, through which new methods, innovations, and improved quality were introduced.

**Implementation:** The GoE remained consistent in priorities and mechanisms over time through which progress has been demonstrated. These have progressively focused on addressing inequities in access.

**Figure 5.**
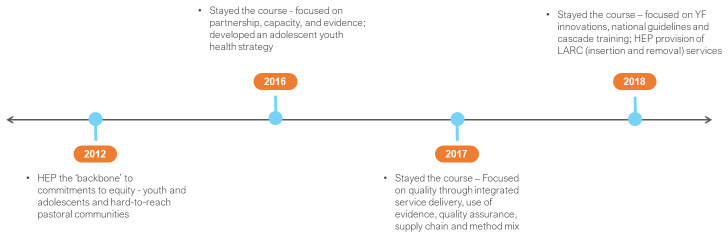
Ethiopia FP2020 commitments and timeline. HEP – Health Extension Programme; LARC – long acting reversible contraception.

Ethiopia’s HEP was the backbone of its FP2020 commitments in 2012 ( Figure 5). The HEP was attributed with improvement to FP indicators that pre-dated FP2020 (mCPR doubled from 2005). Building from this momentum, national commitment focused on addressing the country’s youthful population, through YFS and youth outreach as well as addressing hard-to-reach pastoral communities. The GoE further committed to increasing its budget allocation for FP each year and estimated its funding gap at 50 per cent.

Country actions reported in 2016 emphasized partnership, capacity, and evidence. This included the development of public-private-partnership (PPP) guidelines, integrated service delivery, stronger evidence and use in decision making as well as quality (e.g. related to training, stock outs, and method mix). A national FP-CIP was developed for the period 2015/16–20. As part of its commitment to youth, a 2015–2020 adolescent youth health strategy was developed, informed through research on the factors affecting utilization of FP among adolescents. The GoE reported expenditure for FP during the 2015–2016 fiscal year was estimated at US $27.5 million, inclusive of human resources.

In 2017, as part of FP2020 revitalized commitments, the GoE stayed its programmatic course. In 2018, YFS innovations were reported that expanded access for FP at universities, technical and vocational institutions, and youth centres. National guidelines and cascade training on YFS were implemented while implant and IUCD insertion and removal were added to the basket of contraceptives offered by health extension workers (HEWs). The GoE continued to track its financing commitments using the Ethiopian national health accounts (NHA).

The Ethiopia case study exemplifies consistency of commitment to a national mechanism (the HEP) and its adaptation to address underserved populations. Commitments emphasized government policy and plans, and positioned FP as integral to the health sector and its transformation. In contrast, the FP-CIP did not appear central to FP2020 updates nor did some FP-CIP interventions, such as a TMA plan, feature at all. Like other countries, there was emphasis on youth-friendly corners in every hospital, health centre, and health post coupled with training on YFS. There was no mention of progress in relation to these. Partner initiatives were not referenced (one exception only) in FP2020 updates compared to other FP2020 country reporting. This did not diminish the importance placed by the GoE on partnership.

### Malawi: *National commitment demonstrates “projectisation” of mechanisms and implementation*

**Contextual factors:** There was Government of Malawi (GoM) recognition of the developmental needs of youth and adolescents with the goal of “no parenthood before adulthood.”

**Mechanisms:** Youth and adolescents were the center piece of the GoM FP2020 commitments. These included intersectoral action and standalone YFS to address the needs of youth and adolescents. There was also emphasis on data and partnership to improve accountability and deliver impact.

**Implementation:** Implementation was not uniform and heavily reliant upon partner resources and support. These tended to focus on project commitments, with implications for sustainability and government stewardship of national policy.

**Figure 6.**
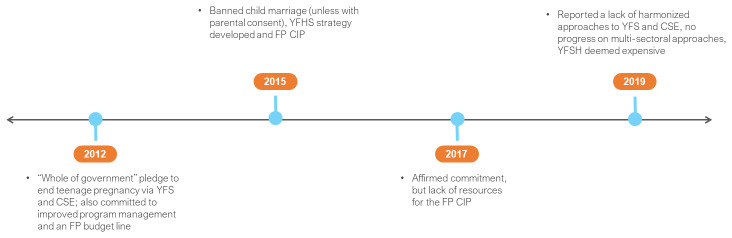
Malawi FP2020 commitments and timeline. YFS – youth friendly services; CSE – comprehensive sexuality education; YFHS – youth friendly health services; CIP – costed implementation plan.

In 2012, the GoM pledged a *“whole of government”* developmental approach to youth, with the goal of *“no parenthood before adulthood”* ( Figure 6). In addition to the provision of youth and adolescent friendly SRH services, the GoM sought to end child marriage by increasing the age of marriage to 18 and introducing comprehensive sexuality education (CSE) for in and out of school youth. The FP program further committed to the introduction of a FP budget line, improved commodity forecasting and data management, strengthening PPP and accountability.

GoM progress was reported through self-assessment in 2014 and 2015. In 2014 the FP2020 update indicated that a partner had successfully advocated with the GoM for the FP budget line (credit for this achievement was not given to the GoM itself). In 2015, the GoM also succeeded in banning child marriage; however, the effect of this law was diluted as girls under the age of 18 could still legally marry if they had parental consent. 2015 also heralded the development of a national FP-CIP (2016–2020) and a stand-alone Youth Friendly Health Service (YFHS) strategy.

Subsequent revitalised commitments in 2017 affirmed the GoM’s initial commitments but noted limited resources for their implementation. In 2019, it was reported that there was a lack of harmonization between partners – *“every partner has their own CSE/life skills curriculum”* – which was viewed as a deterrent for some age groups accessing services. Multi-sectoral investments in the YFHS were also reportedly slow to materialize, *“due to lack of understanding of the interconnectedness amongst the different sectors.”* While no midterm review of the YFHS was conducted due to funding, issues with its effectiveness started to emerge:

- YFHS accreditation focuses mainly on structures and not necessarily on how services were delivered, which increased the likelihood that provider attitudes remained poor.
- There was limited dissemination of key strategic documents to frontline service providers.
- Some accredited facilities were not maintaining the quality of services to meet the minimum standards and there was poor monitoring of accredited facilities.

Beyond operational challenges, the YFHS approaches used were deemed expensive.

There were also reported challenges with tracking implementation of the national FP-CIP. Estimated expenditure was available but did not align to FP-CIP thematic areas, making it difficult to identify which areas were underfunded. As a result, the GoM recommended the use of institutionalised approaches to budget execution, using national health accounts (NHAs), and not FP-CIP tracking. It was reported that the FP budget line grew progressively from 2014 and in 2017 FP expenditure was estimated at US $ 6.4 million dollars.

The GoM provided detailed and thoughtful responses to all FP2020 reporting requests. These showed that there was strong government commitment to multi-sectoral action under the banner of no parenthood until adulthood. However, sectoral and partner approaches were not always linked to the national FP-CIP and FP2020 commitments. This led to the “projectization” of government mechanisms, and their inefficient and “haphazard” implementation. The YSHS did not appear to draw from the wider global evidence base, which has highlighted the limitations of standalone centres and peer educators/community-based distributors ^11^. Malawi is not unique in this regard as other FP2020 countries have struggled to effectively reach adolescents.

### Rwanda: *National commitment to integration, capacity building, and program sustainability through strong management and mechanisms*

**Contextual factors:** The Government of Rwanda (GoR) placed heavy emphasis on equity and decentralized access as well as program financial stability as part of its FP2020 commitments.

**Mechanisms:** The GoR used existing national mechanisms to improve decentralised access, with a well-defined set of objectives and responsibilities. There was a methodical approach to integration of HIPs such as post-partum FP within government systems.

**Implementation:** There was heavy emphasis on the use of data and evidence to monitor FP2020 progress. This included a costing study to inform planning and budgeting of the scale up of post-partum FP. The GoR also integrated FP in the health insurance scheme to address affordability for clients at point of delivery.

**Figure 7.**
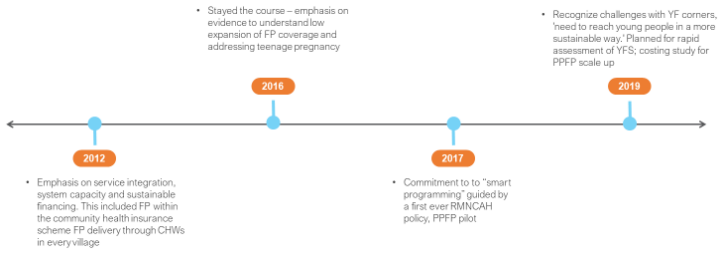
Rwanda FP2020 commitments and timeline. CHWs – community health workers; RMNCAH – reproductive, maternal, newborn, child and adolescent health; PPFP – post-partum FP; YF – youth friendly; YFS – youth friendly services.

In 2012, Rwanda’s FP2020 national commitments were underpinned by the core principles of *“integration of service provision, the increase in healthcare capacity, and the attainment of sustainable funding sources”* ( Figure 7). At the time, the GoR noted the *“adoption of a multitude of national and international development frameworks”*, adapted to the Rwandan context. These included healthcare reforms such as the establishment of a community health insurance scheme ( *mutuelle de santé*), and a system of cooperative-financed community health workers (CHWs) in every village. These initiatives as well as high level political support saw mCPR quadruple from 2005 to 2010, rising from 10 to 45 per cent ^iv^.

Building on this momentum, emphasis was placed on decentralised access, ensuring the availability of FP services in each of Rwanda’s 14,841 administrative villages through 45,000 CHWs, coupled with informational campaigns and demand creation, and the introduction of LARCs and high quality integrated FP services in every hospital and health centre. The Rwanda FP Strategic Plan 2012–2016 noted:

*“While structural changes in health care and supply chains have led to noteworthy improvements in FP and other services, there are still many challenges that must be overcome. As such, a strategic plan is needed to coordinate FP efforts around a well-defined set of objectives and responsibilities.”*

In 2016, the FP2020 country action plan emphasized evidence, to understand the low expansion of the coverage of FP services. It was noted at the time that there were concerns with CHW capacity, health worker bias, financial instability of the health system, and a rise in teenage pregnancies. The 2018–2019 country acceleration and action plans committed to *“smart programming”* guided by a first ever RMNCAH policy. Emphasis was placed on *“cost-effective implementation…an integrated and coordinated approach to facilitate harmonization of activities among partners and systematic monitoring for greater impact.”* As part of a more integrated approach - and to address missed opportunities for FP - post-partum FP was to be scaled up in all health facilities. Along with scale, choice and quality were emphasized. 

The 2018 self-assessment reported on some delays with the finalization of the RMNCAH policy as well as the need to merge previously standalone strategies for FP and adolescent sexual and reproductive health (ASRH), ensuring that *“no critical elements are left behind.”* Through its emphasis on evidence, such as a FP barriers study that was conducted during this period, the GoR reflected that “y *outh friendly corners are still few and scattered in health facilities which needs much attention, new innovative strategies in order to reach young people in a sustainable way.”* GoR reported progressive improvement with post-partum FP, estimated to have improved from 1 to 47 per cent over a two-year period in the ten pilot districts. To facilitate scale up to all of Rwanda’s 30 districts, a costing study was commissioned. This suggested that post-partum FP was affordable, estimated as US 0.10 per capita for the first year of intervention. The GoR also integrated FP in the health insurance scheme to address affordability.

The Rwanda case study exemplifies consistency in national commitments to expand access to FP. The program drove a strong push for review and analysis and used this to inform and monitor interventions. Emphasis on scale did not diminish a desire for quality implementation. Partners were aligned behind FP goals and key practices, under the stewardship of the Ministry of Health, which was also horizontally accountable to the Prime Minister’s office. A singular commitment to post-partum FP showed that this practice could be affordably scaled (it is the only FP2020 country included in the case studies to have done this). The GoR recognized the limitations of some of the YF approaches being used and the need to find more sustainable and scalable models.

### Ouagadougou Partnership (OP) countries: *Ambitious regional commitments and focus on high impact practices with less attention to implementation*

**Contextual factors:** In the selected OP countries, there was elevation of FP as a development objective, in the context of the demographic dividend. Insecurity also featured in several OP contexts creating access and financing challenges for FP service delivery. 

**Mechanisms:** There was heavy emphasis on policy and financial commitments that included complex multi-sectoral action, e.g. demographic dividend (all countries) and unfunded policy objectives such as free FP services (Burkina Faso). These did not fully account for contextual constraints.

**Implementation:** There was projectisation of HIPs, which were mainly reliant on implementation through partners. Given wider health systems weaknesses and access constraints, there was evidence of implementation languor.

**Figure 8.**
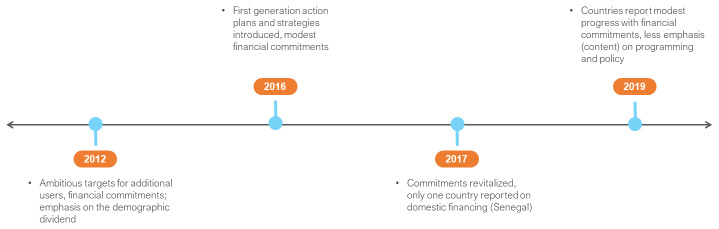
Ouagadougou Partnership FP2020 commitments and timeline.

The francophone countries of Senegal, Burkina Faso and Niger were all FP2020 commitment makers in 2012. At the time, the three countries placed a high premium on reducing total fertility and increasing mCPR, setting ambitious contraceptive prevalence and user targets. This emphasis was in line with their membership in the Ouagadougou Partnership (OP) ( Figure 8), which sought to reach at least 2.2 million additional users of FP methods in ten countries ^v^. Senegal, Burkina Faso and Niger also set ambitious financial commitments. For example, Senegal sought to increase the government’s commodity budget by 200 per cent and double the overall budget for the management of the FP program. Burkina Faso was reported to *“push domestic resource mobilisation to unrivalled levels”* ^12^ within the region as part of its goal of free provision of contraceptive services and products for all clients.

National ministries of health shepherded FP action plans and strategies; these tended to be *“first generation”* and recognised systems constraints and other contextual barriers. For example, countries emphasised task shifting, the inclusion of the private sector and community-based distribution, in their programs and strategies. There was also recognition of the needs of adolescents, in the context of early marriage, high parity and poor birth spacing. Other HIPs adopted were outreach, with an emphasis on LARC, and social and behaviour change communication, to foster demand and community acceptance of FP.

The private sector was a revenue source to be tapped – for example, Niger looked to the private sector for financial support implementing its national health strategy. The World Bank also afforded a revenue source. In Burkina Faso and Niger, amongst other Sahelian countries, the Sahel Women’s Empowerment and Demographic Dividend (SWEDD) project coincided with FP2020 ^vi^. This project propelled FP into the development realm, ensuring that FP was represented in multi-sectoral coordination units, often under the ministry of finance or planning (from 2018, these reportedly evolved into demographic dividend observatories). 

In 2016, the three countries developed FP2020 action plans, which provided a stock taking of opportunities, challenges, and priorities. It was reported that the government of Niger had allocated CFA 200 million (US $ 345,000) annually for the purchase of contraceptive consumables from point of commitment in 2012. However, from 2015, government financing was reduced, and reallocated to security, given heightened concerns and costs associated with this. In Senegal, the contraceptive budget was reported to have increased from 100 million CFA (US $172,000) to 300 million (US $516,000) in 2016. Burkina Faso reported that with a change in government, domestic financing for FP was reduced. Despite this situation, the push for free provision of FP continued, building on the precedent of free MCH services, which could accommodate post-partum FP.

In 2017, the three countries revitalised national commitments; this reaffirmed the social and economic positioning of FP as a tool for the development of human capital. It also affirmed OP country commitment to increase national FP budgets by 10 per cent per annum to 2020. However, only Senegal reported on its financing in 2017, which increased to 500 million CFA francs (US $860,000) and was reported to have increased further in 2018.

In 2018, Niger’s self-assessment reported that it had released 62 million FCFA or US $100,000 for consumables, with a second tranche expected while the rest of the country assessment featured partner updates. In 2018, Burkina Faso reported domestic resources of 1.3 billion CFA francs (US $673,000) for FP. The country also reported extensive advocacy with sub-national authorities for the development of budget lines, of which 20 of 93 authorities did so with varying amounts allocated. Burkina Faso remained committed to free FP; it reported that the costing was approved, but that this would require resource mobilisation, with plans to advocate for FP inclusion in the national health insurance after 2020.

The Senegal, Burkina Faso and Niger case study exemplifies an emphasis on policy and financial commitments with less attention to delivery mechanisms. Action plans and self-assessments focused on domestic resource mobilisation and policy, with less attention placed on programmatic updates. Complex policy processes and multi-sectoral arrangements featured from 2012 and dominated the FP2020 reporting landscape to 2020. While these have the potential to be transformative, there was evidence of implementation languor – despite the adoption of an array of HIPs, there was little sense of progression. 

## Conclusions

*Have you ever done everything right in a development program — followed every technical best practice — but still missed the mark? ^13^*

All national FP programs included within the country comparative analysis benefitted from their engagement with FP2020. However, not all were able to convert FP2020 national commitments into national ownership. Critical factors emerging from the case study analysis suggest the following:

- **Context:** Country programs that adapted HIPs through thoughtful selection, regular monitoring, and course correction, were more responsive to context and better able to scale interventions.
- **Mechanism:** Country programs that embedded commitments within national health reforms and transformative agendas were able to sustain commitment and mechanism more effectively over time. In other countries, where there was greater variation in mechanism, commitment fluctuated. There was less sense of momentum and greater reliance on external “forces” (i.e. projects and partners).
- **Implementation:** Country programs that were able to balance central coordination with devolved implementation, more effectively translated commitments to action. Country programs that demonstrated strong program stewardship were able to convert partner resources into national commitments, reducing “projectization”.
- **Monitoring:** Country programs that placed emphasis on monitoring of progress to inform plans and course correct, were better able to steward national commitments and partner inputs. Additional forms of analysis, such as the costing study in Rwanda, provided further evidence on a HIP, in this case post-partum FP, and plans for scale up.

Two phenomena emerge from the analysis that merit reflection:

- **Isomorphic mimicry:** the over reliance on pre-designed solutions, where country programs adopt form but not function, often at the behest (and good intentions) of partners. This may be one reason that best practices and FP-CIPs do not translate into impact in all contexts. Greater understanding of where and why these practices are effective may be helpful for country programs as they consider future commitments.
- **Maslow’s hammer**: the over reliance on available tools and methodologies. An example that emerged from the case studies was reliance on youth friendly standalone centers and corners as the favored tool to address youth and adolescents within a range of contexts. While there is evidence to show that these approaches are not effective, they continue to be applied.

In many FP2020 contexts, there is less need for a technical “hammer” and greater need for engaging politically on sensitive issues that constrain women’s and adolescent empowerment and rights and access to FP. The India case study exemplifies this, but other country programs skirt around this fundamental issue. National commitments would benefit from greater anchor in transformational policy and practice outcomes (particularly in contexts where ASRH policies languor in draft form or on shelves). This work needs a wide aperture and tactical engagement. This may also require being more directive in policy priorities as partners – and national FP program managers - may shy away from more politically oriented work, preferring to stay focused on technical “hammers”.

## Data availability

All self-assessments and action plans can be found on the FP2020 website under government commitment makers and the respective country.

## Footnotes

^i^ Of note, FP2020 countries are not consistent in the use of FP2020 reporting tools by year. This tends to vary by region with more tools completed for sub-Saharan African country programs.

^ii^ Financed through the Kreditanstalt für Wiederaufbau (KfW), the OBA program provided subsidized high-quality safe motherhood, clinical FP and gender violence recovery services to a population of approximately three million poor people in selected districts (now counties) in Kenya over four phases, from 2006–2017.

^iii^ These were a signature of the Emergency period in the late 1970s when male and female sterilisation camps were used to meet contraceptive targets.

^iv^ The Rwanda government’s Economic Development and Poverty Reduction Strategy called for an increase the mCPR to 70% by 2016.

^v^ In addition to Senegal, Burkina Faso and Niger, the other OP countries include Benin, Cote d’Ivoire, Guinea, Mali, Mauritania and Togo.

^vi^ SWEDD was approved in 2014 and is due for completion in 2023.
