# Supplementary material for: National ownership of family planning: What do FP2020 commitments have to do with it?
Source: Gates Open Res. 2021 Feb 24;5:25. [Version 1] doi: 10.12688/gatesopenres.13225.1 (PMC8175870; doi:10.12688/gatesopenres.13225.1)
Supplement: Peer review history

# Peer review: Reviewer response for version 1

**DOI:** 10.21956/gatesopenres.14448.r30583

**Published:** 2021-06-03

**Authors:** Michael Mbizvo

**Affiliations:** 1 Population Council, Lusaka, Zambia

The authors provide interesting insights related to FP2020 commitments, based on country case studies and related outcomes. These are analysed in terms of policy, financial and political commitments. Critical factors are analysed in relation to responsiveness to contextual needs, scalability of interventions, potential sustainability, and subsequent ownership. While benefits of engagement with FP2020 are identified, and elaborated, the authors note that not all have been able to translate the FP2020 commitment to national ownership. Whereas reference is made to the need for engaging, politically, on issues that constrain access to family planning by women and adolescents, in the FP2020 context, additional insights for the future could include alluding to the potentially negative implications for such failure, both in terms of health and achieving national development targets. While political and program leadership could be changing in countries, over time, the rationale and factors that underlie the commitments, in the first place, may not be as apparent, especially if investment in simultaneous research and strong monitoring systems were not part of the commitments.

Is the work clearly and accurately presented and does it cite the current literature?

Yes

If applicable, is the statistical analysis and its interpretation appropriate?

Not applicable

Are all the source data underlying the results available to ensure full reproducibility?

Yes

Is the study design appropriate and is the work technically sound?

Yes

Are the conclusions drawn adequately supported by the results?

Yes

Are sufficient details of methods and analysis provided to allow replication by others?

Yes

Reviewer Expertise:

Reproductive health sciences, epidemiology, HIV, adolescents’ sexual and reproductive health, family planning, andrology.

I confirm that I have read this submission and believe that I have an appropriate level of expertise to confirm that it is of an acceptable scientific standard.

# Peer review: Reviewer response for version 1

**DOI:** 10.21956/gatesopenres.14448.r30450

**Published:** 2021-03-08

**Authors:** Sarah Castle

**Affiliations:** 1 Sarah Castle Consultancy Ltd, London, UK

The paper, which is very clear and well-written, uses mini-case studies to offer insights to discuss a number of factors shaping the implementation of FP2020 country commitments. It draws on data from country-status updates including country self-assessments, action and acceleration plans and revitalised commitments of national FP programmes published on the FP2020 website.

 The analysis explores factors relating to context, mechanisms, implementation and monitoring and provides detailed case studies for a number of national settings with a variety of contraceptive prevalence profiles as well as financing structures. It concludes that, in general, they benefited from their association with FP2020 but that although technical intervention was important, there was also, in many cases, a significant need for engaging politically with the socio-cultural contexts which frame women’s rights and empowerment which also strongly influence family planning uptake.  

 The paper would have benefited from more discussion around who is best placed to engage with the rights-based discourse - the answer being civil society organisations. In addition, although specific attention is given to adolescents, in many national contexts, married adolescents are often under-served and particularly hard to reach. Plans and strategies need to take account of prevailing socio-cultural factors which often require this group to ‘prove’ their fertility upon marriage thus making family planning uptake extremely low. In addition, it should be acknowledged that a country’s profile can evolve during the duration of the CIP and affect both family planning demand and service delivery - for example, COVID-19 has now often drastically impacted service provision and use. In other settings, such as some of the Ouagadougou Partnership counties, insecurity, and associated gender-based violence has increased significantly in recent years raising the demand for comprehensive sexual and reproductive health services, including emergency contraception. In the light of this, there may not be a ‘one-size fits all’ in relation to the indicators used to assess country performance and these may need to be adapted to reflect local contexts and priorities. Within-country variation, particularly, in financing is well-addressed in the paper and notes differences in, for example, the dominance of partner-led funding in Pakistan compared with domestic financing in Kenya. Learning around the efficacy and challenges associate with different service delivery mechanisms could maybe benefit from more south-to-south exchange and comparison among the FP2020 countries - for example, a sharing of learning around Ethiopia’s HEP programme and its associated task-shifting emphasis at the community level. Similarly, the implementation, costing and impact experiences of the scaling-up of up post-partum family planning in Rwanda could be shared to benefit other national contexts.

 As FP2030 takes shape and new Country Implementation Plans are created and influenced, the paper has great relevance which could serve to make them more efficient, cost-effective and relevant.  

Is the work clearly and accurately presented and does it cite the current literature?

Yes

If applicable, is the statistical analysis and its interpretation appropriate?

Yes

Are all the source data underlying the results available to ensure full reproducibility?

Yes

Is the study design appropriate and is the work technically sound?

Yes

Are the conclusions drawn adequately supported by the results?

Yes

Are sufficient details of methods and analysis provided to allow replication by others?

Yes

Reviewer Expertise:

Family planning, sexual and reproductive health, adolescent reproductive health, health systems, human resources for (reproductive) health.

I confirm that I have read this submission and believe that I have an appropriate level of expertise to confirm that it is of an acceptable scientific standard.
